# Study supports for rural mature-aged university health students: a Stakian multicase study

**DOI:** 10.1186/s12909-024-05128-4

**Published:** 2024-02-20

**Authors:** Claire Quilliam, Nicole Crawford, Carol McKinstry, Alison Buccheri, Sara Brito

**Affiliations:** 1 The University of Melbourne, 49 Graham Street, Shepparton, VIC 3630 Australia; 2 Curtin University, Kent Street, Bentley, WA 6102 Australia; 3 La Trobe Rural Health School, La Trobe University, Edwards Rd, Bendigo, VIC 3552 Australia; 4 Violet Vines Marshman Centre of Rural Health Research, La Trobe University, Edwards Rd, Bendigo, VIC 3552 Australia; 5 Occupational Therapy Department, Federation University, Northways Rd, Churchill, VIC 3842 Australia; 6 Occupational Therapy Department, Eastern Kentucky University, 521 Lancaster Ave, Richmond, KY 40475 USA

**Keywords:** University, Mature-aged student, Education, Rural populations, Workforce recruitment, Learning support

## Abstract

**Background:**

The participation and success of university health students in rural areas is critical in addressing the maldistribution of the rural health workforces internationally. Particular attention to the experiences of mature-aged health students is needed to build a sustainable rural health workforce, given the higher proportions of mature-aged university students in rural, regional and remote areas compared with metropolitan areas and rural mature-aged students wanting to stay in their communities. However, little is known about the kinds of supports rural mature-aged students require to succeed with their studies.

**Methods:**

Drawing on rural standpoint theory and using structural inequality as a retention lens, we explored the current and potential supports that rural mature-aged nursing and allied health students require to successfully participate and complete their pre-professional university course. A Stakian multicase study was undertaken with cases at three rural university campuses in Australia. The data collection was primarily qualitative, with semi-structured interviews, campus surveys and focus groups involving 36 participants (including students, academic and professional staff, and placement supervisors).

**Results:**

This study found supports were provided formally and informally by the university, by the community and manifested by students. Several support gaps as well as potential supports to alleviate them were identified. These include formally acknowledging the mature-aged cohort and their diverse experiences and non-university commitments; fostering connections between mature-aged students; making university affordable; preparing mature-aged students for university; adapting course content and delivery; and restructuring placements for mature-aged students.

**Conclusions:**

We argue that rural mature-aged nursing and allied health students require supports that are age-specific, appropriate to the community context, and harness existing relational processes of rural university campus activity. Rural university campuses need to involve rural mature-aged students and other stakeholders relevant to each context in the process of identifying and implementing student supports for this cohort.

**Supplementary Information:**

The online version contains supplementary material available at 10.1186/s12909-024-05128-4.

## Background

Enabling rural people to participate and succeed in higher education remains an international challenge, despite efforts to achieve universal access to higher education [1]. Educational disparities are complex, with rural people in high- and low-income countries facing a range of challenges, including accessing higher education within or close to their rural community, complicated processes to migrate to metropolitan areas, and a lack of policies to promote equitable access to higher education [2–4]. Some rural people, particularly mature-aged people, are often not able to move to metropolitan campuses to undertake their degree of choice. This results in a narrow selection of course options for those fortunate enough to have access to higher education within or close to their community [5]. The ongoing challenges rural people face to attain health qualifications need addressing when health workforce shortages in rural communities and poor health outcomes for rural people persist [6].

Retention strategies can aid participation of rural students experiencing challenges in higher education [7]. Retention strategies often reflect deficit thinking based on Bourdieu’s [8] cultural capital, where non-traditional students, such as rural mature-aged students, are expected to develop capital and *assimilate* into academic life [9]. Instead, Naylor and Mifsud [9] argue universities and other societal structures need to turn inward and examine how they can adequately support these students to succeed in their studies. In this study, we present the findings from a multicase study in Australia on the types of supports available to and needed by rural mature-aged nursing and allied health university students to successfully participate in their studies and join the rural health workforce.

Numerous terms are used to define students who come from or reside in regional, rural, and remote areas. In Australian higher education research, the term *regional and remote student* is often used to encompass regional, rural, and remote students.^1^ However the Modified Monash Model (MMM) describes seven *rurality* categories, which is frequently used in the field of Australian rural health [11]. In this article, the terms *rural students* and *rural campuses* are used due to the rural location of cases involved in this study. Given the lack of consensus relating to the definition of mature-aged students in higher education, in this study, a *mature-aged student* is considered as being twenty-one years of age or older at commencement of their undergraduate course [10]. Mature-aged students are also referred to in the literature as older students, mature learners [12], adult learners [13] and non-school leaver students [14].

### Rural health workforce development

Providing equitable access to healthcare for rural people is challenging. Access to healthcare in rural settings is impacted by the availability of qualified health professionals, among other factors [15]. Having sustainable rural health workforce development and thus services for rural healthcare is influenced by access to higher education in rural areas [16]. Health professionals in high-income countries often need to hold university-level pre-registration qualifications (e.g., [17]), although enabling access to health professions education, particularly in rural areas, is a complex matter requiring collaboration between a range of government bodies, education institutions and health peak bodies [18].

Several approaches have been used to address the maldistribution of the rural health workforce. These include encouraging and/or mandating largely metropolitan-based health students to complete rural clinical placements (also known as work-integrated learning or fieldwork), and encouraging metropolitan and international-origin health professionals to develop community connections in their current rural work locations [19, 20]. Over the last two decades, health workforce policy internationally has advanced to recognise and improve access to health professions training through a *pipeline* approach, where health courses are delivered to selected students in their rural community [21, 22]. While the pipeline approach tends to favour medicine, the positive impact of this approach can be applied to nursing and allied health disciplines [6, 23]. Nursing and allied health professionals form a critical part of the rural health workforce. In rural communities, nursing and allied health professionals may be the only health professionals providing services [24].

The pipeline approach to workforce development acknowledges the role and strength of place and belonging in education attainment and career development for rural people. A recent examination of the rural health workforce literature found evidence of *sense of place*, and *place attachment*, but not *belonging-in-place* concepts [25]. However, belonging-in-place ought to be of interest to those exploring sustainable rural health workforce mechanisms. It refers to experiences that stretch beyond presence in a community or the act of merely living or working in a community, as some health professionals who move to a rural community experience [26]. Belonging-in-place is achieved over time through a range of ongoing engagements [27], including social and environmental interactions [25, 28]. Drawing on these concepts, we propose that people already living in rural communities are likely to have stronger belonging-in-place in their respective communities than health professionals who travel or relocate to a rural community to perform their job. Identifying rural cohorts who could contribute to the rural health workforce, and meeting their educational support needs could help to address longstanding issues with rural health workforce attraction and retention. This approach aligns with Roberts’ [29] rural standpoint theory that emphasises the capacities of rural people to address rural issues, drawing on rural knowledges and processes.

### Rural mature-aged students

Mature-aged people in rural communities could be considered a cohort with potential to make a greater contribution to the rural health workforce [30]. Rural mature-aged people who decide to study often remain in their rural communities because of their multiple attachments to place [2, 31]. Mature-aged people bring a range of life experiences and knowledges to higher education and have non-university commitments that are typically different to school-leaver students, including caring responsibilities and financial burdens [30]. However, the literature describes very few supports specifically for mature-aged nursing and allied health students enrolled on rural campuses, particularly at a university or government-level [32]. Rural communities have capacity in their unique histories, community connections, skillsets, and local infrastructure to offer rural mature-aged students a range of study supports [31]. The provision of suitable study supports for rural mature-aged higher education students could significantly impact the sustained growth of the rural health workforce.

### The Australian context

In Australia, there is notable disparity between the educational attainment of people living in metropolitan and rural areas [33]. This educational disparity in Australia reflects international trends that impact healthcare worldwide. In Australia, over one-quarter of the population live in regional, rural and remote communities, which are characterised by diverse geographies [34]. Current higher education policies recognise the importance of providing suitable supports for higher education students in rural areas, as demonstrated by the rise in Regional University Study Hubs (RUSHs)^2^ [7, 35]. However, higher education course offerings in rural areas are limited compared to those in metropolitan areas [36]. Nursing and allied health university courses are available in some rural communities, although mostly in the more highly populated eastern seaboard states. Rural people under 35 in Australia are less likely to have a higher education qualification compared with their metropolitan counterparts, and, as such, feature in the current major review of higher education in Australia [33, 37]. Mature-aged students form a sizeable proportion of the student population in, and from rural areas in Australia [30].

## Methods

### Aim and objectives

The aim of this research was to explore study supports for rural mature-aged nursing and allied health students. The research question was: What study supports do rural, mature-aged nursing and allied health students require to successfully participate and complete their pre-professional university course? Specifically, we wanted to a) describe the context in which study supports are manifested, b) identify the types of study supports currently available and gaps in support, and c) identify potential study supports to enable rural, mature-aged nursing and allied health students to successfully complete their studies and join the rural health workforce.

### Study design

Stake’s [38] instrumental, multicase study methods guided this qualitative study. Instrumental case study is used to explore the particularities and differences between multiple cases to answer an overarching research question. Stakian case study research is underpinned with a constructivist epistemology, where knowledge is considered to be constructed by those involved in the research process, rather than being objectively *found* [39]. Consistent with constructivist epistemology, Stakian case study research captures and illustrates multiple realities within and across cases [40]. Constructivist epistemological and relativist ontological lenses were appropriate to capture multiple perspectives on supports for rural, mature-aged students across different rural communities. The University of Melbourne (2023-21707-40631-8), La Trobe University (2022-21707-24802-6) and Federation University (E22-001) human research ethics committees provided approval to conduct the study. Hyett’s [41] case study criteria, adapted from Stake [38], informed the study design (see Additional file 1).

### Case selection

Three rural university campuses were selected as the cases, based on three criteria: a) university campus located in rural Victoria, Australia, b) university campus offering nursing and/or allied health pre-registration courses, c) campus capacity to participate in the study. Each case comprised the rural university campus in its entirety: its physical presence in the community, courses offered, students, staff (academic and professional), placement supervisors, and campus events. Authors used personal networks to identify potential cases and invite campus leaders, via email, to involve their campus in the study.

### Data collection

Survey, interviews, and focus groups were used to develop a comprehensive description of each case from multiple perspectives. Participants included professional and academic staff working at the campuses, mature-aged students enrolled in nursing or allied health courses, and placement supervisors. Data were collected between February and November 2022, virtually or via telephone due to transient COVID-19 pandemic government and university enforced restrictions.

Participants were recruited using electronic methods, including email and videoconference during student classes. Snowballing methods were then used to recruit participants. Campus leaders distributed research project information to relevant course coordinators, academic and professional staff, and invited staff to forward to colleagues and students. Potential participants were provided with a plain language statement and consent form via email.

Two key theories informed the design of data collection. Rural standpoint theory [29] informed the development of data collection tools. Survey, interview and focus group questions about campus descriptions and existing study supports aimed to capture rural knowledges and processes from those who are familiar with and involved in the participating cases. Structural inequality [9] also informed the development of data collection tools, particularly interview and focus group questions about studying experiences, gaps in supports and potential study supports. These questions aimed to elicit data indicating potential strategies for higher education providers, governments, and other key stakeholders to consider in future policy development. The survey questions captured 1) campus characteristics, including number of health courses, 2) student characteristics, and 3) current students supports including access to a campus librarian. Student interview questions focused on experiences of studying on the campus, knowledge of supports and support gaps, and suggestions for potential supports. University staff interview questions focused on experiences of working on the campus and supporting mature-aged students. Placement supervisor interview questions focused on experiences supporting mature-aged students while on clinical placement. Interviews were audio recorded, transcribed verbatim, deidentified and checked by participants for content accuracy. Campus leaders were asked questions tailored to their leadership role and knowledge of campus functions. During the focus groups, participants discussed campus characteristics, study experiences, and reflected on current and potential supports that were identified through the survey and interview process. Focus groups were audio recorded and transcribed verbatim. Identifying information was removed from focus group transcripts and checked by participants for accuracy.

### Data analysis

*Within-case* analysis included two phases, as per Stake’s [38] method. Phase 1 involved initial analysis of data, where authors reflected on the data, particularly how it answered the research questions, and how their own assumptions impacted their reactions to the data. Phase 2 involved Saldaña’s [42] first cycle coding methods: descriptive, in-vivo and process coding. Authors identified and agreed on themes and sub-themes comprising the codes. Case reports were developed for each campus.

*Cross-case* analysis followed a mixed approach as described by Miles and colleagues, where case-oriented and variable-oriented analysis approaches are combined [43]. The authors used case-orientated strategies to identify particularities and similarities across campus characteristics. Drawing on the case reports, short vignettes were developed to capture key particularities of each case, and a short narrative to capture similarities and differences across cases. Variable-orientated strategies were then adopted. Codes relating to current and potential supports were aggregated in a concept table using the stacked comparable cases approach to compare codes across cases. Categories of supports sharing patterns or similarities were identified and re-organised in the table to illustrate these coding categories (see Additional files 2 and 3). Following this stage, the authors reflected on the vignettes, and the aggregation of current and potential supports and asked three questions offered by Miles and colleagues [43]: 1) “why does variability exist in these data?; 2) what specific conditions might have influenced the variability?; and 3) in what ways might this variability influence and affect other outcomes and consequences?” (p. 167). A short narrative was written in response to the questions to summarise the cross-case analysis. The data collection and analysis processes were iterative in nature and involved constant reflexive discussion. Drawing on a constructivist epistemological lens allowed the authors to capture a wide variety of knowledges on student supports, rather than to identify one truth as is common with typical triangulation processes [44].

### Reflexivity statement

The principal researcher, CQ, is a rural researcher who completed their PhD at a rural campus as a mature-aged student with two young children and supported their partner to complete a social work pre-professional qualification as a mature-aged student. NC has taught university students from diverse and underrepresented backgrounds; informed by a constructionist epistemology, she primarily conducts qualitative research with interests in student equity in higher education. CM is a registered occupational therapist and academic involved in overseeing a range of health courses delivered on regional and rural campuses. AB is a rural speech pathologist and researcher with experience supervising mature-aged allied health students. SB completed her pre-professional qualification as a mature-aged student and is an experienced qualitative researcher who lives and works rurally as an occupational therapist. Throughout the conception and completion of the study, team members discussed their personal and professional backgrounds, life experiences, including rural life, and acknowledged the impact of these on their perceptions of the study, areas of importance to investigate, and concepts identified in the data.

## Results

The study involved 36 participants in total, including nine university staff, six placement supervisors, and 21 students (see Table 1). One electronic survey per campus was completed by campus leaders. Thirty-three semi-structured interviews ranging in duration between 30 and 80 min were conducted with participants across the three cases via videoconference (case one: *n* = 14; case two: *n* = 11; case three: *n* = 8). A focus group was conducted with students for each case ranging in duration between 57 and 70 min, outlining the preliminary interview and survey findings for students to provide feedback (case one: *n* = 6; case two: *n* = 2; case three: *n* = 2).

**Table 1:** Demographics of study participants

	Case one	Case two	Case three
**Number of students**	10	7	4
**Age category on enrolment:**			
** 21-30yrs**	2	1	1
** 31-40yrs**	5	4	2
** 41-50yrs**	2	2	1
** 51-60yrs**	1	0	0
** 61-70yrs**	0	0	0
**Number of university staff**	3	3	3
**Role:**			
** Professional (including campus leaders) **	2	2	2
** Academic **	1	1	1
**Number of placement supervisors**	2	2	2

Vignettes of the three cases (referred to as case one, case two and case three) that describe the context where study supports are manifested or provided to rural, mature-aged nursing and allied health students are now presented, followed by the current and potential supports described by participants (see Table 2 for further case characteristics).

### Case characteristics: vignettes

The campus of case one is well-established in a large rural town over 150kms from the nearest city. The campus has tailored its offerings to meet changing local industry needs over time, which are currently education and health. Recent demand for allied health workforce in the area led to the development of new facilities and course offerings that have attracted local and metropolitan students. Collaborations with local health and education providers have resulted in multiple pathways for health courses, including via the local Technical and Further Education (TAFE)^3^ provider courses and work-integrated-learning with local health services. Students complete their studies via a range of modalities, including attending classes on-campus, online and via a RUSH.

The campus is small compared to typical metropolitan university campuses and this was described as both beneficial and challenging for students. Smaller class sizes enable teaching staff to be accessible and approachable, allowing staff to check in with students and facilitate access to supports when required. Access to staff is deemed important by this campus’ mature-aged students, whose non-university commitments often changed unexpectedly during the semester. Reduced access to resources and facilities resulted in the campus having limited capacity to meet the diverse needs of its students, particularly students with disabilities. From the student perspective, the campus lacks adequate space for eateries and a bookshop, which prevents mature-aged students meeting with other mature-aged students beyond their cohort. In case one, mature-aged students represented 58.6% of all nursing and allied health students enrolled at the campus in Semester 1, 2022.

The campus in case two was established in the 1990s, in a large rural town approximately 400kms from the nearest city. There is a history of the campus providing education to produce local health professionals rather than the community attracting external health professionals, due to the geographical distance from metropolitan areas. Participants explained that community members rarely want to move away to study, and it is particularly challenging for mature-aged students with non-university commitments in the community to travel for study. Long term retention of the health professionals moving to the community is limited.

The campus was originally developed with the aim of meeting the higher education and workforce needs of the local community. Courses currently offered at the campus reflect industry demands, including pre-registration nursing and social work courses, although participants noted that many community members are unaware of the campus’ existence and what it offers. While the strong demand for nursing and social work professionals contributes to high employment rates following graduation, campus leaders and academic staff acknowledged that the opportunity to work in community service organisations without a formal qualification can negatively impact on student attraction and retention at the campus, particularly in the social work courses. The local TAFE provides pathways into courses at the campus, although some students commence their university studies as a mature-aged student with industry experience and previous university qualifications.

There are strong connections between staff and students, between students, and between health professionals, health services and campus staff. The campus was described by staff as welcoming, although it experiences challenges in providing the full complement of resources and supports compared to metropolitan campuses. Vacancies in support staff, insufficient student numbers to sustain clubs and societies and a lack of basic on-campus resources (e.g., student kitchen facilities) were identified as frequent challenges. The variable internet and telephone connectivity in the rural community impacts online learning and support services. In case two, mature-aged students represented 58.8% of the total pre-registration nursing and allied health students enrolled on this campus in Semester 1, 2022.

The case three campus was established in the early 1990s in a large rural community approximately 200kms from the nearest city. Historically, the campus has always aimed to improve access and participation of local people in higher education, and at meeting local workforce needs. The campus offers courses that respond to local industry demands, including those for health and education. The campus is well connected with the local community and frequently hosts important events.

The existence of the campus in the community has improved access to higher education for students who are the first-in-family to participate in university, and who may not have considered university study otherwise. For students to fully participate and succeed, staff have adopted an approach that includes providing pastoral care along with delivery of course content. With this approach, staff—both academic and professional—typically form strong relationships with students and work alongside other staff in a wide variety of roles.

The sustainability of course offerings and student services are described as challenges for the campus. The COVID-19 pandemic restrictions created further challenges, resulting in a workforce change where university staff roles transitioned from a focus on local students to a university-wide focus, working beyond the campus itself. Students access services that use generic emails and contact details and seek support from a non-identifiable team located elsewhere, rather than approaching a staff member on the campus. Since COVID-19, staff have found it difficult to engage with students. For example, online students, who previously might have attended the campus to study, are less likely to do so now. In case three, mature-aged students represented 57.9% of the total pre-registration nursing and allied health students enrolled on this campus in Semester 1, 2022.

**Table 2:** Case characteristics

Campus characteristics	Cases		
	Case one (% total campus student number)	Case two (% total campus student number)	Case three (% total campus student number)
**Students (total)**	1593	231	326
**Mature-aged students enrolled in pre-registration nursing and allied health courses**	481 (30.2%)	50 (21.6%)	70 (21.5%)
**Students from a low-socioeconomic status background**	362 (22.7%)	166 (71.9%)	215 (66.0%)
**Students with disability**	241 (15.1%)	34 (14.7%)	48 (14.7%)
**Students who identify as having Aboriginal and Torres Strait Islander background**	25 (1.6%)	15 (6.5%)	17 (5.2%)
**Students who identify as having a non-English speaking background**	196 (12.3%)	10 (4.3%)	38 (11.7%)
**Students who are first-in-family to attend university**	804 (50.5%)	Not reported	160 (49.1%)^a^
**Total number of university staff**	231	28	25
**Pre-registration nursing and allied health course disciplines**	Physiotherapy, Occupational Therapy, Speech Pathology, Nursing, Midwifery	Social Work, Nursing	Social Work, Nursing

^a^Case three number of students who are first-in-family to attend university is an estimate

### Current supports

A wide range of supports for students were identified across the three cases, including those that were informally and formally provided by the university, community-provided and student-provided. Twenty informal university-provided supports were identified (See Additional file 2 for the list) and were organised into five categories: i) Campus culture, including the environment being inclusive and friendly; ii) Interpersonal supports, such as academic staff reaching out to struggling students and being available for a chat; iii) Course supports, including academic staff supporting students to submit assignments; iv) Referring and providing services, such as professional staff referring students to services, and; v) Placement adaption supports, including allowing students to have input into placement allocation. Some informal supports, such as academic staff being family friendly (supporting students with young children/babies to attend class), were specific to case two. Other informal supports were consistent across cases, particularly with interpersonal supports such as academic staff checking in with students, developing rapport with students, and referring students to other supports. A student explained:

I didn’t actually kind of even approach her [lecturer]. I think she just approached me one day. She’s like, you know, ‘what’s going on? How are you? I’ve noticed you were quieter today’. … And um, I was kind of like, ‘oh yeah…’ I kind of spoke to her, and she was like, ‘Oh, well why don’t we try this, or why don’t you try that?’ …She doesn’t have to [do this]. She could have just packed her books up and walked out. …like most lecturers would. And it’s not in her job description, you know, she’s not going to get paid to do any of that. (case three, student, participant 9)

Sixty-four formal university-provided supports were identified (see Additional file 2 for list). These were organised into six categories: i) Characteristics of the campus itself, including the location and existence of the campus; ii) General administrative supports, such as information technology support and student advisors; iii) Academic supports, including access to librarians and foundation studies programs; iv) Wellbeing and social supports, such as a crisis telephone line and accessibility supports (i.e., learning access plans); v) Financial supports, including university scholarships, and; vi) Placement supports, including placement liaison staff.

There were significant commonalities in formal supports across cases, particularly with financial, general administrative, and academic supports. Some participants in case three explained that the campus offered wellbeing and social events, including those that would appeal to mature-aged students, such as ‘bring your child to university’ day and an engagement program for online students. However, other student and staff participants noted that COVID-19 had impacted on the availability of several formal case three supports:

I’m more online and more involved with students from other campuses now, whereas pre-COVID, [my role] was really just focused on this campus. …Since COVID our students um … just aren’t on-campus as much… Before [COVID-19] I would have offered students classes, you know, showing them how to search, how to find information, how to reference, all of that sort of thing. And I would have done them face-to-face. But now, we offer them online, which, I mean, it’s a good thing; it does offer more opportunities for the students to attend at a time that suits them, because it’s shared with staff from other campuses, so we offer a lot more. … But at the same time, I’m not getting that contact that I used to with our students. (case three, staff, participant 25)

Across all cases, students who participated in the focus groups were not aware of several university-provided supports identified in relation to their campus. These participants described many supports as being uncommon, dependent on relationships on the campus, or university-wide supports that were mainly accessible for students on metropolitan campuses: “A lot of the supports that the uni talks about offering or talks about existing really only occur, like for [the city campus]” (case two, student, focus group).

Of the formal university-provided supports, no supports were specifically provided for mature-aged students. The generic nature of supports provided by university campuses reflects an explicit assumption held by university staff in cases one and three (and implicit in case two), that mature-aged students do not need or want supports specifically tailored to their cohort: “We don’t tend to say ‘we’re going to offer this [support] for just mature-aged students’… because, you know, a lot of them [mature-aged students] probably don’t want to be focused on as something different” (case three, staff, participant 25). Mature-aged student participants explained that generic supports were inherently tailored to students with different non-university commitments to mature-aged students, such as full-time school leaver students. They described the need for supports that were specific or more suitable for them as mature-aged students:

There’s a lot of stuff the uni does that I look at it and I’m like, ‘Oh, this is such a 19-year-old thing or such a 20-year-old thing.’ Like, the clubs and societies are all for children, it feels like. … So, I guess that’s an area they could expand in. (case one, student, participant 14)

Twenty-nine community-provided supports were identified (see Additional file 2). These were organised into six categories: i) Placement preparation supports, including using community connections to provide flexible placements; ii) Host organisation supports, such as the provision of student placement orientation videos; iii) Placement supervisor supports, including making students feel like part of the health team during placements; iv) Employer and work colleague supports, such as healthcare colleagues offering feedback; v) Financial and information supports, including government funded scholarships, and; vi) Community services, such as after school care. Scholarships funded by the government departments and other sources external to universities were common across the three cases, although there was significant variance across the cases in other support categories. One community-provided support specifically provided to mature-aged students was placement supervisors writing grants for mature-aged students. Student placement accommodation and after-school care were only mentioned in case one. In case two, unique supports were described that enabled placement opportunities to be developed specifically for local students, drawing on community connections:

What happens is the student placement team for [the university]; they work in [another town], so they’ve got no idea personally who the students are, and they just allocate them out to [host organisation] places…. If there’s any issues, the students get in touch with the placement team and say, ‘Hey’, you know, ‘these are my issues around the placement’ … then the placement team will try and work [a solution] with the hospital. But they don’t… yeah, it’s not a personal approach. …I just take the personal approach… Most of the time I know them because I’ve taught them at uni… [I] just call them [the student] up and say, ‘What’s going on? What can we help with?’ (case two, supervisor, participant 23)

While this support was identified by a case two placement supervisor, case two student participants were not familiar with this support, which suggests access to it depends on relationships between students and university staff and placement supervisors, as stated by one student: “The placement supports: I haven’t felt that at all, unfortunately” (case two, student, focus group).

Twenty-nine student-provided supports were identified (see Additional file 2) and organised into four categories: i) Individual strategies, including choosing work shifts to fit with study commitments and maintaining physical activity; ii) Peer supports, such as mature-aged students supporting each other via study groups, iii) Partner supports; and iv) Immediate and extended family supports, including supportive extended family members. Peer supports were common across cases, particularly mature-aged students supporting each other by sharing information and resources online and via informal study groups:

It’s not just academic, it’s also emotional/social… When we’re together in person and can actually see each other, [and] support each other. … [We send] each other ideas on how to study it in a different way. I’m finding that [mature-aged student] support is a lot better than what the uni is offering [laughs]. …Mature-aged support group [is good] because there’s nothing like that. (case one, student, participant 8)

### Potential supports

Across the three cases, participants identified significant support gaps and 96 potential supports to address these (see Additional file 3 for the list). Potential supports were organised into seven categories: i) Acknowledge the rural, mature-aged student cohort, by, for example, increasing mature-aged student committee representation; ii) Foster connections between mature-aged students, by organising mature-aged student social events, iii) Make university more affordable for mature-aged students, through local health services financially supporting students; iv) Prepare mature-aged students for university and workforce entry, by for example, facilitating health professionals’ mentoring of mature-aged students; v) Adapt course content, including recognising prior learning/life experience by providing exemptions for relevant core subjects or an entry point for mature-aged students; vi) Develop inclusive teaching and learning practices, for example, provide flexibility in scheduling/timetabling, and; vii) Enhance placement success for mature-aged students, including sufficient planning for flexible placements, among other potential supports.

There were commonalities in potential supports across cases. Participants from all campuses suggested universities could go further to acknowledge the prominent existence of the rural, mature-aged student cohort and draw on the knowledge of this cohort to identify and implement suitable supports, facilitate connections between mature-aged students across courses, and make university more affordable for mature-aged students. Building on the relational nature of supports described in case three, student participants emphasised the need for age-specific social supports, particularly the opportunity for mature-aged students to build additional social connections with other mature-aged students in other disciplines and year levels:

Bringing together the mature-aged students of other cohorts [would be good]. … [I’m] just kinda sitting there by myself, like looking at people. …Whereas, if you’ve got someone there… just makes you feel a little bit more, um, at ease or at home or, like, comfortable. (case three, student, participant 9)

One major gap in current supports noted by student participants was around supporting mature-aged students through clinical placements, considering mature-aged students typically have additional non-university commitments to juggle during placement:

[For] every single mature-age student that has come through, the issue has been affordability. …Like, by far… It is a major issue for them because a lot of these mature-age students are working part time as well. So, um, [one student] … had to take long service leave from his job in order to get that [final placement completed]. (case two, supervisor, participant 33)

Staff, students, and supervisor participants noted that significant effort would be required to restructure placements to suit mature-aged students, consistent with the potential placement supports described. Proposed changes included improving communication between stakeholders such as professional peak bodies and health services to support placement flexibility in length, hours and mode, providing financial support, implementing internship-based placement models, and providing additional supports prior to and during placements to ensure suitability for mature-aged students. Ultimately, having locally available placement planning supports for mature-aged students was considered potentially beneficial by students, staff and supervisors: “The placement liaison [person] to be on-campus: that would be a game changer” (case two, student, focus group).

Case one student participants identified several potential supports around adapting course content that were not identified by participants in case two and case three. This variance may be due to several case one student participants having extensive previous tertiary-level experience. These students felt their prior knowledge was overlooked. They were frustrated with spending limited study time on subjects where they felt they already had sufficient competency, for example around interpersonal communication: “I really think that they need to take our life experience more seriously and give us more recognition for it” (case one, student, focus group). These participants were confident they had adequate knowledge and skills to meet a higher entry level and called for this prior knowledge to be recognised, by provision of exemptions for relevant subjects for mature-aged students:

The content that I need to know is drowned down by all this group work… There should be an entry point where we can actually go: ‘Here, here and here is where I’ve done it in my previous degrees. I’ve been successful in the workforce.’ There needs to be a mature-aged entry point. (case one, student, focus group)

## Discussion

This multicase study aimed to explore the study supports required for rural mature-aged nursing and allied health students to successfully participate and complete their pre-professional university course. The findings identified a plethora of supports offered on three rural campuses in Victoria, Australia, and potential supports to address support gaps. The range of supports identified reflects the diversity of students studying at these campuses and communities in rural areas, addresses a gap in the literature around supports for rural mature-aged nursing and allied health students [32], and offers higher education providers, rural communities, and governments new strategies for supporting rural people to complete their studies and join the rural health workforce. Consistent with Naylor and Mifsud’s [9] structural focus, the findings emphasise the role of rural campuses and staff, the centrality of relationships in current supports, and the need to consider community, relational and age-specific features to support rural, mature-aged nursing and allied health students to complete their studies and join the rural health workforce.

The existence and importance of rural campuses formed a common thread in all three cases. Having a physical campus made pursuing a rural health career possible for mature-aged students who were not able to travel or move to the closest metropolis for their studies. The findings suggest many mature-aged students benefit from face-to-face engagement and other supports delivered on-campus. Providing higher education health courses in rural areas helps overcome longstanding patterns of workforce maldistribution by allowing rural people to gain qualifications to join the rural health workforce, although this approach requires sustained investment and commitment from all stakeholders [6]. To optimise support provision, rural campuses could provide suitable study supports for mature-aged students in partnership with local learning centres, schools and libraries [31].

Relationships between universities and the community, students and staff, staff and rural health professionals, students and family and peer student relationships were described as critical by participants for student access to suitable supports. These relationships formed the basis of many informal-university supports, community and student-provided supports, and were a major strength of the participating rural campuses. Students felt these relationships were key to developing a sense of belonging, as reported by Hays [45]. Having a safe connection to draw on when non-university commitments impinged on allocated study time or adjustments to study load were required, was important. In all cases, academic staff were described as using a relational approach, developing rapport with students beyond what was expected because they appreciated that these efforts were helpful in providing other supports to students and covering support gaps on rural campuses.

The existence and importance of relationships on rural campuses reflects the concept of *dual relationships* in rural contexts [46]. From a rural standpoint, these relationships inherently provide complex connections through and within the community and tie higher educational experiences to place [29]. These complex relationships are in constant flux, impacted by local and external influences on the provision of student support. In case two, community connections were used to provide flexible placements for students, although these were provided in an ad-hoc manner when student need was evident via other engagements and a placement supervisor had discretionary capacity to meet the need. In case three, local supports were impacted by the removal of campus-specific student support roles in favour of a university-wide support approach. This change removed the place-based relationship from the support process, which was described as valuable by participants. Our findings suggest that the presence of on-campus services provide important relational supports for rural mature-aged students, and access to suitable supports that often depend on student-staff relationships and knowledge held by local campus staff. Rural campuses and their broader universities need to harness the relational approach and adopt systems that allow these supports to be experienced more consistently for rural mature-aged nursing and allied health students.

Mature-aged students represented approximately half of all pre-registration nursing and allied health students studying at rural campuses involved in each case, which is consistent with prior research [10]. Rural mature-aged nursing and allied health students are therefore an important cohort in respect to understanding study experiences and perspectives on study supports when developing sustainable rural health workforce mechanisms. Gray et al. [47] reported that rural mature-aged students typically described having different non-university commitments to school-leavers. Like Gray [47], our findings demonstrate how these commitments are closely tied to the nature of supports considered important to participate and succeed at university. However, in all three cases, the supports provided to students were *generic* in nature and aimed at meeting a wide variety of non-traditional student needs.

Mature-aged students identified support gaps in several study aspects including clinical placements, finance, and social connections. For example, participants across all cases stressed that scholarships were rarely fit-for-purpose for mature-aged students because they were seldom available to part-time students or not tailored to contemporary rural mature-aged study costs such as childcare and travel expenses. Some formal supports advertised as available were not actually available to or suitable for mature-aged students on rural campuses. Consistent with the findings of Crawford and Emery [30], the findings of this study illustrate that in practice, many generic supports were implicitly designed for the *traditional student*—the full time, metropolitan-based school leaver, and were often unsuitable for rural mature-aged students.

With rural campuses having a high proportion of mature-age students, further exploration is warranted into how supports can be better designed and provided for these students without disenfranchising other student cohorts. The findings of this study suggest that underpinning the design and delivery of supports is a tension between the ideas of *universal* and *tailored design*. Universal design, usually discussed in relation to students with disability [48], was a concept typically discussed by staff participants in this study, where it was suggested all students should have access to a range of supports, regardless of their characteristics and support requirements. This implies that study supports should be suitable for mature-aged students, despite not being created specifically for them. The need for tailored designed supports, suggested by student participants, recognises the unique experiences of mature-aged students. Tailored supports have been successfully implemented for other student cohorts. Williams [49] described culturally affirming practices for Black students who are first-in-family to attend university and have a low-income status. This illustrated the importance of knowing and meeting the support needs of non-traditional students in a strengths-based manner. There are benefits from designing supports for a range of students; however, our study findings suggest ongoing reflection may be required to ensure supports tailored specifically for student cohorts do not revert to focusing on the traditional student.

The findings of this current research suggest that mature-aged students would benefit from being considered an identified cohort with a diverse range of life experiences, non-university commitments, and study challenges, and with a need for specific supports during their studies. Rural campus leaders (and senior university leaders) may need to purposefully curate a selection of supports for rural mature-aged nursing and allied health students, considering the diversity of experiences and characteristics of this cohort. Given the overlap of characteristics in non-traditional students, specific mature-aged student supports would likely benefit students from several equity or equity-like groups. The diversity of potential supports identified in each case of this study indicates new supports need to be specific to campus contexts, considering local community resources and student needs. Rural mature-aged students are well positioned to identify pressing support needs in their context.

### Strengths and limitations

This study has identified a range of study supports for rural mature-aged nursing and allied health students that have the potential to support the development of the rural health workforce, and in turn, the health of rural communities. The supports identified in this study are specific to the geographic, cultural, and economic contexts in which the case study campuses are situated. As noted by participants, supports are not always provided systematically, but in response to situated relationships, local knowledge, and other resources. The finding that rural mature-aged health student supports need to be carefully crafted for each context holds significance for rural campuses beyond Australia, particularly in other high-income countries where access disparities exist alongside resources [50]. Future research on supports for rural mature-aged health students could compare the identification and implementation processes in different countries to better understand similarities and differences in processes across contexts. Future research could also explore the impact of providing context-specific study supports on workforce retention in the rural mature-aged nursing and allied health graduate cohort from a range of perspectives, including community and industry.

Due to the COVID-19 pandemic restrictions, data collection methods were limited to using electronic means and field observations could not be conducted. Future research exploring the merit of supports for rural mature-aged students would benefit from field observations, to capture the relational richness on rural university campuses, and its impact on student participation and success.

## Conclusions

Mature-aged students form a significant proportion of the rural health university student cohort and offer untapped potential to contribute to the rural health workforce. This study identified current and potential supports for mature-aged nursing and allied health students studying on three rural university campuses in Victoria, Australia. Mature-aged nursing and allied health students have significant non-university commitments that impact the nature of supports required to participate and succeed in their studies. Given the potential contributions that mature-aged students could make to the rural health workforce and to the health of rural people, rural university campus leaders should consider implementing age-specific and contextually appropriate supports, in partnership with mature-aged students and local community stakeholders.

### Supplementary Information

**Additional file 1.**

**Additional file 2.**

**Additional file 3.**

## Data Availability

The datasets generated and/or analysed during the current study are not publicly available due to not having consent from participants to publish but are available from the corresponding author on reasonable request. Data can be found in this published article and in additional information files.

## Abbreviations

MMM: Modified Monash Model
RUSH: Regional University Study Hubs
TAFE: Technical and Further Education

## Footnotes

1 For further explanation of the terms and definitions, refer to Crawford [10].

2 RUSHs are physical learning centres where higher education students studying online and living in rural areas can study with reliable internet and computers, and academic and pastoral support.

3 In Australia, TAFE is one type of vocational education and training, with funding from state and territory governments.
