# Supplementary material for: Study supports for rural mature-aged university health students: a Stakian multicase study
Source: BMC Med Educ. 2024 Feb 20;24:163. doi: 10.1186/s12909-024-05128-4 (PMC10880340; doi:10.1186/s12909-024-05128-4)
Supplement: Supplementary file 1 — Additional file 1. [file 12909_2024_5128_MOESM1_ESM.pdf]

Additional file 1

To be read alongside article “Study supports for rural mature-aged university health students: a Stakian multicase study” published in BMC Medical Education.

Authors: Quilliam, C., Crawford, N., McKinstry, C., Buccheri, A., & Brito, S.

Additional file 1 provides supplementary information regarding Hyett’s (1) strategies to enhance rigour in case study research, adapted from Stake (2).

Table 1. Hyett's (1) Strategies used to enhance case study rigour, adapted from Stake (2).

Checklist criterion	Strategy/example
**1. Is the case adequately defined?**	Used Stake's (1995) understanding of context features to consider in case studies: historical, temporal and spatial, economic, political, social and personal, cultural (p. 43).
**2. Is there a sense of story to the presentation?**	Case vignettes and survey, interview and focus group data are used to explain how student supports are experienced on the three campuses.
**3. Is the reader provided some vicarious experience?**	The case vignettes and participant quotes are provided for the reader.
**4. Has adequate attention been paid to various contexts?**	Three rural campuses were explored in Victoria, Australia.
**5. Were data sources well-chosen and in sufficient number?**	Participants were selected according to stakeholder role (student, staff and placement supervisor), discipline, and student year level.
**6. Do observations and interpretations appear to have been triangulated?**	Involving students, campus staff and placement supervisors in interviews, focus groups and campus surveys offered insight into multiple perspectives on student supports.
**7. Is the role and point of view of the researcher nicely apparent? Is empathy shown for all sides? Are personal intentions examined?**	Researcher personal reflections on approach to data collection and was conducted during initial data in line with Stake's phase 1 data analysis approach.

References

1. Hyett N. Exploration of international case studies on community participation and health. Bundoora, La Trobe University 2016.
2. Stake RE. The art of case study research. Thousand Oaks, CA: Sage; 1995.
