# Supplementary material for: Study supports for rural mature-aged university health students: a Stakian multicase study
Source: BMC Med Educ. 2024 Feb 20;24:163. doi: 10.1186/s12909-024-05128-4 (PMC10880340; doi:10.1186/s12909-024-05128-4)
Supplement: Supplementary file 2 — Additional file 2. [file 12909_2024_5128_MOESM2_ESM.pdf]

Additional file 2

To be read alongside article “Study supports for rural mature-aged university health students: a Stakian multicase study” published in BMC Medical Education.

Authors: Quilliam, C., Crawford, N., McKinstry, C., Buccheri, A., & Brito, S.

Additional file 2 provides supplementary information regarding current supports identified by participants.

Current supports identified by participants

Table 1. University-provided supports (informal)

Categories	Case one	Case two	Case three
**Campus culture**			The campus atmosphere/environment: being inclusive and supportive, friendly
**Interpersonal supports**	Academic staff building rapport to enable timely supports	"It's that relationship that we build with them": maintaining strong relationships with students	Academic staff knowing students, understanding their circumstances and noticing their challenges
		Academic staff being family friendly (supporting students with young children/babies to attend class)	
	Academic staff being approachable and responsive, having one on one chats: "make myself available"	"You don't let someone you know struggle": Academic staff reaching out to struggling students and being available for a chat	"They will often be the person that a student will talk to first": Academics talking with students and providing pastoral care
**Course supports**	Academic staff providing assignment due date extensions and special consideration support	Academic staff coordinating changes to study for students facing personal issues	Academic staff using their discretion around rules—e.g., provide extensions because they know the students' circumstances
	Academic staff supporting students to submit assignments	Academic staff supporting students to catch up on content	Academic staff providing useful feedback on assessments
**Referring and providing services**	Academic staff being referral agents	Academic staff referring students to services	Academic staff referring students to relevant services
	Academic staff providing mental health services	Professional staff referring students to services	
**Placement adaption supports**		Allowing students to have input into placement allocation	

Table 2. University-provided supports (formal)

Categories	Case one		Case two		Case three	
**Campus characteristics supports**	Campus location	Small class sizes in small rooms	Creating flexible and adaptive curriculum		Existence of the campus: provision of physical study place	Relatively smaller class sizes—staff and students get to know each other
	Delivery of coursework in regional hospitals	Blended learning	Blended delivery			
	Regional University Study Hub location	Opportunity to study part-time			Pathways to university: e.g., being able to articulate from a TAFE nursing course to a university course	
**General administrative supports**	IT support	Case management	IT support		Guides/FAQs provided online	
	Peer administration support		Student advisors		Student advisors (including a Student experience advisor)	Student administrative support
					On campus childcare	Careers event (prior to COVID-19): Careers

						dinner including local industry stakeholders and students
**Academic supports**	Library access	Writing feedback program	Library consult supports	Studiosity	Librarian	
	Preparatory program (offered to commencing students)	Foundation studies program (taken 6 months before course commencement)	Transition course		Transition program for nursing TAFE students articulating to university	Extra-curricular events for students (e.g., mental health first aid course)
	Peer-facilitated subject support groups	Industry entry academic sessions	Study sessions	Assignment and exam extensions	Sessions for specific courses—e.g., librarian attending nursing classes	
	Learning skills advisors	Online academic information	Academic supports (previously knowns as the learning hub)	Peer learning advisors	Academic skills support	Peer learning advisors
**Wellbeing and social supports**	Counselling services	Crisis telephone line	Wellbeing services (counselling)	Wellbeing events	Counselling services	Social events (prior to COVID-19): e.g., a “Bring your child to uni” day

	Disability support officers (learning access plans)	Aboriginal and Torres Strait Islander support workers	Accessibility supports (learning access plans)		engagement program for online students	Industry connections for future employment
			Social events		Peer leadership: Student association	
**Financial supports**	Financial services	Scholarships	Finance supports	Scholarships	Food programs	Grants/bursaries
**Placement supports**	Placement location special consideration	Student grants			Placement liaison person	

Table 3. Community-provided supports

Categories	Case one	Case two	Case three
**Placement preparation supports**	Local hospital providing future workplace opportunities via placement	Using community connections to provide flexible placements	Support of local health providers (taking students on placement)
	Student placement accommodation	Taking a "personal approach" to organising placements	
**Broad host organisation supports**	Host organisations working with universities to manage placements	Fostering good communication with mature-aged students on placement	Support from the health service while on placement
	"Trying to get them through": providing flexible placement days	Student orientation video	
		Initial placement conversations about support	
		Taking a personal support approach to supporting students *during* placement	
**Placement supervisor supports**	"Giving them opportunity": supporting mature-aged students to learn	Placement supervisors writing grants for mature-aged students	Supportive staff while on placement (e.g., supervisor)
		Placement supervisors providing emotional support on placement	Debrief sessions/check-ins (while on placement)
		Making students feel like part of the team	
**Employer and work colleague supports**	Employer flexibility	Supportive employer	Students having a workplace supportive of them studying at university (e.g., can take study leave from job to undertake placement)
	Healthcare colleagues offering feedback		
	Healthcare services providing employee		

	sponsorship		
**Financial and information supports**	Grants offered external to the university	Government funded scholarships	Government funded placement website
		Facilitating internships around placements	Bursaries for placements (via external sources)
**Community services**	After school care		

Table 4. Student-provided supports

**Categories**	**Case one**		**Case two**		**Case three**	
**Individual strategies**	Using social media to plan study activities and manage non-university responsibilities	Choosing work shifts to fit study commitments	Study space		Maintaining physical activities	Time management
					Negotiation to manage placements	Maintaining home life
**Peer supports**	Mature-aged students supporting other by communicating and sharing strategies (mostly online)		Mature-aged student connections	Online social media student discussion	Mature-aged students supporting each other (e.g., understand each other; pick up each other's children from school; swap work shifts with each other).	Mature-aged students are a resource—e.g., provide information about their course to each other (especially for students who missed orientation or classes)
	Mature-aged students supporting other via mature-aged students study group	Discipline-specific support communities	Discipline-specific social groups (initiated and facilitated by students)		Study groups	Student association: Social work club (run by mature-aged students)
	Mature-aged students supporting each other on placement	Friends (outside of university) as support		Connections between diverse students (school leavers and mature-aged) within disciplines	Peer support while on placements	Mature-aged students mentoring younger students
**Partner supports**	Partner support		Supportive partner			
**Immediate and extended family**	Family support		Supportive extended family		Family support	

**supports**	"Showing them anything is possible": Illustrating academic success to children	Children providing encouragement
