# Supplementary material for: Study supports for rural mature-aged university health students: a Stakian multicase study
Source: BMC Med Educ. 2024 Feb 20;24:163. doi: 10.1186/s12909-024-05128-4 (PMC10880340; doi:10.1186/s12909-024-05128-4)
Supplement: Supplementary file 3 — Additional file 3. [file 12909_2024_5128_MOESM3_ESM.pdf]

Additional file 3

To be read alongside article “Study supports for rural mature-aged university health students: a Stakian multicase study” published in BMC Medical Education.

Authors: Quilliam, C., Crawford, N., McKinstry, C., Buccheri, A., & Brito, S.

Additional file 3 provides supplementary information regarding potential supports identified by participants.

Potential supports identified by participants

Table 1. Potential supports for mature-aged nursing and allied health students studying at rural campuses.

Categories	Case one			Case two			Case three			***Exemplar quotes***
**Acknowledge the rural, mature-aged student cohort**	Enrol more mature-aged students	Acknowledge mature-aged students juggle responsibilities in course planning	Increase mature-aged student committee representation	Draw on mature-aged student knowledge to improve student supports	Provide childcare		Support mature-aged students by increasing staff awareness of their circumstances	Offer information or events for their family/children to feel a part of university life	Raise awareness for lifelong learning opportunities/professional development	*Maybe having a specific seat or couple of seats for someone who is mature-aged [on the student board] or even having a separate mature-aged student [working group], whatever, would work. … Because then you're getting that that voice from different… someone who's mature-aged, along with someone who's just younger and coming out of high school… (case one, student, participant 8)*<br><br>*Maybe childcare for some of the students 'cause I know a few, they have kids and sometimes if there's no-one to look after the kids at home they cannot come to school… or the clinic. So I think that's a very good idea. Like on campus childcare. (case two, student, participant 21)*<br><br>*I think if the students' families feel like they're part of it or feel supported in some way then the students are gonna be able to achieve much more, you know, able to be successful. (case three, student, participant 9)*
	Ensure mature-aged students understand course workload	Develop university employee module on mature-aged student study experiences	Recognise mature-aged student intent				Re-introduce face-to-face supports that existed prior to COVID-19 restrictions, but have since moved online	Employ staff based on the Campus who work specifically with the Campus students	Ensure supports are regionally centred, rather than designed for metropolitan students and contexts	
	Re-market part time study						Ask students what supports they need			
**Foster connections between mature-aged students**	Develop mature-aged student social networks	Establish mature-aged study support technology networks	Foster parent study group development	Offer mature-aged student social events	Provide interdisciplinary and cross-year social events	Establish peer mentoring	Tailor social events for rural, mature-aged students (as opposed to duplicating events designed for	Facilitate connections between mature-aged students in different courses	Provide extracurricular events that foster learning and connecting with peers	*They could even start kind of like… study groups… You know how you like there's the mum and kid fitness classes; but if they had mum and kid study class… Something like that would be awesome, where the kids could*

							school-leaver students in metropolitan areas)			*socialise and go and play, you know, over here and we could like study over here. (case one, student, participant 19)*
	Establish local rural allied health networks									*Some like peer mentoring sort of opportunities... would be nice. I know it's hard... but... whether we could schedule a time to meet together, um, and... meet up with other students, have a chat about their experience, share our experience. And, yeah, especially getting, you know, the older students' perspective on placement and stuff like that. (case two, student participant 13)*
**Make university more affordable for mature-aged students**	Provide adequate financial supports	Local health services financially supporting students	Pay students to join the health workforce	Provide fit-for-purpose scholarship funding (flexible spending)	Offer merit-based scholarships	Provide placement childcare payments	Raise local financial support for mature-aged students	Offer subsidies in the local community (e.g., subsidised gym membership)	Provide on campus financial support staff	*I think, some mature-aged grants and scholarships would be absolutely fantastic.…There are … options for scholarships and things like that, [but] they're generalist kind of ones. They're not um, specifically directed at um, mature-aged students (case one, student participant 8)*
	Develop mature-aged student grant criteria		Create cadetships/ traineeships for rural mature-aged people	Offer paid placements						*1,000 hours of unpaid placement is absolutely brutal. …The financial strain on myself, it's really difficult. And I have a very supportive partner who is covering my part of the rent and everything like that. So I'm really struggling, and I'm in a really privileged position. And I'm providing my free labour to the hospital, I'm holding a caseload. I'm there full-time. And I'm getting nothing. It doesn't have to be a full-time wage, but just something would be a massive help. (case two, student, focus group)*

										[Need to] have … the financial support person, who would be on campus, be able to assist us with those type of scholarship supports because a lot of the time students do need [support]. … I haven't applied for scholarships yet because I just don't know how to (case three, student, focus group)
**Prepare mature-aged students for university and workforce entry**	Offer pre-semester general technology seminars	Develop a rural-specific job website		Offer pre-enrolment education (what to expect at university)	Provide more opportunities to work in healthcare before graduation	Facilitate health professionals mentoring mature-aged students				*Probably looking at opportunities for traineeships for... perhaps your allied health assistant who wants to retrain as an OT, or your enrolled nurse who wants to retrain as an RN, and perhaps is someone who's either in the workplace or in the community... And … that's probably... a bit of a symbiotic approach between the health service who absolutely has a vested interest and then the…education provider as well. (case one, placement supervisor, participant 4)*<br><br>*I think we need to look at some way [of]… making [that information] available to commencing students. … So, you know, they do get their head around… how to connect to [the university internet], making sure their email's set up, making sure they understand how to print on campus. (case two, staff, participant 26)*
	Use suitable support technology			Provide orientation to university logistics	Promote supports more directly	Host career exhibitions				
**Adapt course content**	Deliver content suited to mature-aged career development	Provide additional resources (including utilizing existing university resources) to	Recognise prior learning/life experience by providing exemptions for relevant core subjects or an entry point for							*I would like a little bit more input from staff about directions that the career could take you. … I want to actually see that… career planning and I think that probably is a mature-aged*

		supplement online learning	mature-aged students							*way to think. (case one, student. participant 14)*
	Offer learning tool demonstrations									*I just wonder whether the uni could maybe do a bit more promotion of some of these things [supports]…It might be nice if, you know, [support] people popped in and introduced themselves and, asked if you have questions. (case two, student, participant 13)*
**Develop inclusive teaching and learning practices (delivery)**	Maintain blended subject delivery to suit mature-aged students	Hold classes at times that suit parents	Allow students to choose group work colleagues	Provide course flexibility	Offer micro-credentials	Develop online options for mature-aged students	Offer inclusive assessment deadlines for mature-aged students	Provide flexibility in course offerings	Provide flexibility in scheduling/ timetabling	*I guess just as a mother, having classes that don't go too late [would help]. (case one, student, participant 20)*
	Offer more one-on-one supports	Reduce focus on group work	Organise classes to support mature-aged students to continue health workforce employment	Provide student led extra classes for catching up on content	Offer more flexibility with extensions	Use a collaborative approach to staff supporting students (case management)				*We have a cohort of people who are off working in the field ... but the commitment of they have to sign up for four years full-time or, or whatever – this traditional degree pathway – it's, it's too much for them. But if they could do some micro-credentialling along the way, but in a way that it can be additive and that that'll count towards the next bit, and they can pop in and do a bit, do that, pop out and go back to the workforce, come in again. (case two, staff, participant 28)*
	Reach out further into the region	Provide face-to-face lectures and labs for rural (distance) students		Provide tutors for mature-aged students	Hold specific classes for mature-aged students					*It would be really helpful if, you know, for future quizzes if they could stay open like on a weekend, um, just because I know that I'm not the only person in that situation [being a parent and working]. (case three, student, participant 30)*
**Enhance placement success**	Plan for mature-aged student placements	Support part-time placements	Create shorter placements	Foster a culture shift around placement flexibility	Conduct earlier preparation for flexible placements	Restructure placements	Preparation prior to placement commencemen	Offer additional support during placement	Advocate for student study leave from employment	*Well, give us jobs in the local area, placements in the local area for where we're going to work. In the long-term… help us to get to make*

							t/offer clear expectations			*connections in our local community, so that when we're getting to graduation, we can go to such and such clinic down the road and go, 'I've done my placement here. I know this community.' (case one, student, participant 12)*<br>*I suppose we could formalise that process a bit better in … sending out a survey before they start [placement] about what supports do you need. We could get a little bit better at identifying things earlier rather than on the first day. (case two, placement supervisor, participant 23)*<br>*One of the supports you can give a mature-aged student is study leave in their current place of employment, but not many, some of the agencies don't have very clear policies around that. (case three, placement supervisor, participant 24)*
	Keep placements close to home	Use placements to create local work opportunities for mature-aged local people	Couple mature-aged students on placement	Formalise conjoint nursing appointments	Approach placement stakeholders beyond the university	Establish connections with rural GPs to implement placement vaccination program	Ensure placement supervisors are supported	Provide on campus placement liaison officer	Improve communication between university and health services regarding placement responsibilities	
	Facilitate students bringing families on placement	Create flexible placements hours	Improve university-host organisation communication prior to placement	Keep placement work at the placement site	Fully fund external placements for mature-aged students		Offer financial support for student placements	Implement an internship model		
